# Correction: Clonal Diversity and Clone Formation in the Parthenogenetic Caucasian Rock Lizard *Darevskia dahli*

**DOI:** 10.1371/journal.pone.0100067

**Published:** 2014-06-04

**Authors:** The PLOS ONE Staff

## Notice of Republication

This article was republished on May 21, 2014, to correct an error in the title of the article.

The title should read:

Clonal Diversity and Clone Formation in the Parthenogenetic Caucasian Rock Lizard *Darevskia dahli*

The citation should read:

Vergun AA, Martirosyan IA, Semyenova SK, Omelchenko AV, Petrosyan VG, et al. (2014) Clonal Diversity and Clone Formation in the Parthenogenetic Caucasian Rock Lizard *Darevskia dahli*. PLoS ONE 9(3): e91674. doi:10.1371/journal.pone.0091674

Please download this article again to view the correct version. The originally published, uncorrected article and republished, corrected article are provided here for reference.

## Supporting Information

File S1 Originally published, uncorrected article.
(PDF)

File S2 Republished corrected article.
(PDF)
