# Supplementary material for: Clonal Diversity and Clone Formation in the Parthenogenetic Caucasian Rock Lizard Darevskia dahli
Source: PLoS One. 2014 Mar 11;9(3):e91674. doi: 10.1371/journal.pone.0091674 (PMC3950254; doi:10.1371/journal.pone.0091674)
Supplement: Table S1 — Polymorphisms of three microsatellite loci Du215, Du281, and Du323 for nine genotypes in D. dahli (see Figure 2 ). GATA repeat is designated by the letter A. Numbers before the letter A designate the number of the repeat on each of the two chromosomes that are separated in the Table by forward slash “/”. In concatenated sequences used for parsimonies network reconstruction, the absence of a GATA repeat is designated by dash (−). (DOC) [file pone.0091674.s001.doc]

**Table S1:** Polymorphisms of three microsatellite loci Du215, Du281, and Du323 for nine genotypes in *D. dahli* (see Figure 2).

| Genotype/Locus | Du215 | Du281 | Du323 | The polymorphic sites of the concatenated sequences | | | | | | | | |
| --- | --- | --- | --- | --- | --- | --- | --- | --- | --- | --- | --- | --- |
|  |  |  |  | 1 | 2 | 3 | 4 | 5 | 6 | 7 | 8 | 9 |
| 1 | 9A/10A | 9A/11A | 11A/1A | - | - | - | A | A | A | A | - | A |
| 2 | 9A/10A | 9A/11A | 10A/1A | - | - | - | A | A | A | A | - | - |
| 3 | 10A/10A | 9A/11A | 11A/1A | - | A | - | A | A | A | A | - | A |
| 4 | 9A/11A | 9A/11A | 10A/1A | - | - | A | A | A | A | A | - | - |
| 5 | 9A/11A | 8A/11A | 10A/1A | - | - | A | - | A | A | A | - | - |
| 6 | 9A/10A | 9A/8A | 11A/1A | - | - | - | A | - | - | - | - | A |
| 7 | 11A/10A | 9A/11A | 11A/1A | A | A | - | A | A | A | A | - | A |
| 8 | 10A/10A | 9A/10A | 11A/1A | - | A | - | A | A | A | - | - | A |
| 9 | 9A/10A | 9A/12A | 10A/1A | - | - | - | A | A | A | A | A | - |
| Variability  of the repeat numbers | 9-11/10,11 | 8,9/8-12 | 10,11/1 |  | | | | | | | | |

GATA repeat is designated by the letter A. Numbers before the letter A designate the number of the repeat on each of the two chromosomes that are separated in the Table by forward slash “/”. In concatenated sequences used for parsimonies network reconstruction, the absence of a GATA repeat is designated by dash (-).
