# Supplementary material for: Clonal Diversity and Clone Formation in the Parthenogenetic Caucasian Rock Lizard Darevskia dahli
Source: PLoS One. 2014 Mar 11;9(3):e91674. doi: 10.1371/journal.pone.0091674 (PMC3950254; doi:10.1371/journal.pone.0091674)
Supplement: Table S2 — The population indices of gene diversity for three studied loci in five sampled populations. N – number of alleles, Rs – allelic richness, HE – expected heterozygosity. (DOC) [file pone.0091674.s002.doc]

**Table S2:** The population indices of gene diversity for three studied loci in five sampled populations.

| Locus | Population | Allele (N) | *Rs* | H_E_ |
| --- | --- | --- | --- | --- |
| Du215 | Papanino | 3 | 2.087 | 0.511 |
|  | Dzoraget | 2 | 2.000 | 0.545 |
|  | Dendropark | 4 | 3.453 | 0.647 |
|  | Vaagni | 3 | 2.938 | 0.632 |
|  | Fioletovo | 2 | 2.000 | 0.529 |
|  | ∑ | 5 | 2.676 | 0.563 |
| Du281 | Papanino | 3 | 2.087 | 0.511 |
|  | Dzoraget | 2 | 2.000 | 0.545 |
|  | Dendropark | 3 | 2.600 | 0.574 |
|  | Vaagni | 4 | 2.800 | 0.582 |
|  | Fioletovo | 2 | 2.000 | 0.529 |
|  | ∑ | 6 | 2.220 | 0.520 |
| Du323 | Papanino | 3 | 2.478 | 0.511 |
|  | Dzoraget | 2 | 2.000 | 0.545 |
|  | Dendropark | 3 | 2.853 | 0.611 |
|  | Vaagni | 3 | 2.648 | 0.577 |
|  | Fioletovo | 2 | 2.000 | 0.529 |
|  | ∑ | 3 | 2.813 | 0.598 |
| All loci |  | 14 | 2.570 | 0.560 |

N – number of alleles, Rs – allelic richness, H_E_ – expected heterozygosity.
