# Supplementary material for: Correction: Clonal Diversity and Clone Formation in the Parthenogenetic Caucasian Rock Lizard Darevskia dahli
Source: PLoS One. 2014 Jun 4;9(6):e100067. doi: 10.1371/journal.pone.0100067 (PMC4045937; doi:10.1371/journal.pone.0100067)
Supplement: File S2 — Republished corrected article. (PDF) [file pone.0100067.s002.pdf]



Clonal Diversity and Clone Formation in the Parthenogenetic Caucasian Rock Lizard *Darevskia dahli*

Andrey A. Vergun[1,2], Irena A. Martirosyan[1], Seraphima K. Semyenova[1], Andrey V. Omelchenko[3], Varos G. Petrosyan[3], Oleg E. Lazebny[4], Olga N. Tokarskaya[1], Vitaly I. Korchagin[1], Alexey P. Ryskov[1]*

1 Laboratory of Genome Organization, Institute of Gene Biology of the Russian Academy of Sciences, Moscow, Russia, **2** Department of Organic and Biological Chemistry, Moscow State Pedagogical University, Moscow, Russia, **3** Group of Bioinformatics and Modeling Biological Process, Severtsov Institute of Ecology and Evolution of the Russian Academy of Sciences, Moscow, Russia, **4** Department of Genetics, Kol'tsov Institute of Development Biology of the Russian Academy of Sciences, Moscow, Russia

Abstract

The all-female Caucasian rock lizard species *Darevskia dahli* and other parthenogenetic species of this genus reproduce normally via true parthenogenesis. Previously, the genetic diversity of this species was analyzed using allozymes, mitochondrial DNA, and DNA fingerprint markers. In the present study, variation at three microsatellite loci was studied in 111 specimens of *D. dahli* from five populations from Armenia, and new information regarding clonal diversity and clone formation in *D. dahli* was obtained that suggests a multiple hybridization origin. All individuals but one were heterozygous at the loci studied. Based on specific allele combinations, 11 genotypes were identified among the individuals studied. Individuals with the same genotypes formed distinct clonal lineages: one major clone was represented by 72 individuals, an intermediate clone was represented by 21 individuals, and nine other clones were rare and represented by one or several individuals. A new approach based on the detection and comparison of genotype-specific markers formed by combinations of parental-specific markers was developed and used to identify at least three hybridization founder events that resulted in the initial formation of one major and two rare clones. All other clones, including the intermediate and seven rare clones, probably arose through postformation microsatellite mutations of the major clone. This approach can be used to identify hybridization founder events and to study clone formation in other unisexual taxa.

Citation: Vergun AA, Martirosyan IA, Semyenova SK, Omelchenko AV, Petrosyan VG, et al. (2014) Clonal Diversity and Clone Formation in the Parthenogenetic Caucasian Rock Lizard *Darevskia dahli*. PLoS ONE 9(3): e91674. doi:10.1371/journal.pone.0091674

Editor: Taras K. Oleksyk, Univerity of Puerto Rico at Mayaguez, United States of America

Received June 26, 2013; **Accepted** February 13, 2014; **Published** March 11, 2014

Copyright: © 2014 Vergun et al. This is an open-access article distributed under the terms of the Creative Commons Attribution License, which permits unrestricted use, distribution, and reproduction in any medium, provided the original author and source are credited.

Funding: This study was partly supported by the Russian Foundation for Basic Research (grant No. 11-04-00754-a), the RAS Programs "Molecular and Cell Biology" and "Living Nature," and the President RF Program (grant Nos. 5233.2012.4; 2501.2014.4; 2349.2014.4). The funders had no role in study design, data collection and analysis, decision to publish, or preparation of the manuscript.

Competing Interests: The authors have declared that no competing interests exist.

* E-mail: ryskov@mail.ru

Introduction

Naturally occurring unisexual reproduction is documented in less than 0.1% of all vertebrate species [1]. Among vertebrates, true parthenogenesis has been detected only in squamate reptiles, and especially among lizards [2]. Lacertidae was the first family in which this phenomenon was discovered [3]. All but one [1] of the parthenogenetic species originate from interspecific hybridization between bisexual species. They are normally diploid or triploid and commonly exhibit high levels of nuclear gene diversity because of fixed heterozygosity at codominant loci [4]. Each parthenogenetic species usually consists of several of clonal lineages, possibly caused by either mutation, multiple origins, or genetic recombination [5–7]. It is suggested that clonal diversity can also be correlated with the size of the area of origin [8], distinct ecological conditions of the habitat, and age of parthenogenetic species [9,10].

In 1989, Parker et al. summarized the allozyme variation patterns observed among parthenogenetic species of several genera of lizards and proposed a hierarchical model for their origin, to predict whether they arose from single or multiple interspecific hybridization [10]. They concluded that a unisexual species with a single hybridization origin typically has little variation and usually consists of a widespread common clone with a few rare clones. For species with multiple hybridization origins, the pattern of genetic variation is highly variable, with random allele combinations. At the same time, the nature of the founder event at the origin of unisexual species can vary significantly. In some cases, high levels of genetic diversity in allozymes, combined with low variation in mitochondrial DNA (mtDNA), suggest multiple origins from a geographically restricted sample of females [11–13]. In other cases, in which there is low diversity for allozymes and mtDNA, the origins appear to have been strongly restricted, both numerically and geographically [14,15].

The genus *Darevskia* is of special significance, because these lizards have been the subject of extensive ecological and biogeographical studies [16], and because parthenogenesis has probably arisen several times within the group [17]. This genus includes 17 bisexual and seven truly parthenogenetic, diploid species of hybrid origin [16]. The allozyme patterns observed in the parthenogenetic *Darevskia* species are generally in accordance with Parker *et al.*'s model. Among these species, *Darevskia dahli* has a single origin [18].

D. dahli, which is one of the seven unisexual species, occurs in the southern Caucasus Mountains of Armenia and Georgia [16,17]. Allozyme and mtDNA data analysis identified the sexual parents of *D. dahli* as *D. mixta* and *D. portschinskii* [8,17]. At present,

D. mixta occurs only in Georgia, and *D. portschinskii* is distributed in southern Georgia, northern Armenia, and western Azerbaijan [19]. According to Darevsky *et al.* (1967), parental species are mainly allopatric [19]. In Armenia, *D. dahli* is typically distributed in forest regions at altitudes ranging from 700 to 1500 m [20]. It inhabits moderately dry bedrocks and gorge slopes in the forest zone, and also occurs among stony sites in mountain steppe habitats in formerly forested sites. Although *D. dahli* populations are rather numerous, they are usually isolated from one another. However, some of them can be sympatric with bisexual and/or parthenogenetic lizards of the genus *Darevskia*. For instance, in the Dzoraget region (near Stepanavan), *D. dahli* is sympatric with the bisexual parental species *D. portschinskii* and with parthenogenetic *D. armeniaca*; near Sevkar village, D. *dahli* is sympatric with parthenogenetic lizards *D. rostombekovi* and *D. armeniaca*, and with bisexual lizards *D. raddei*. The interspecific hybrids identified in these zones are infertile triploids [21]. In the Papanino population, two distinct color varieties of *D. dahli* are known [16], suggesting the possibility of two distinct clones in this taxon. They differ in belly coloration, which can be yellowish or bright yellow [20]. Some differences between these morphological forms have been observed regarding ecology and egg-number production. Bright-yellow lizards produce fewer eggs than do pale-yellow lizards, and they tend to be less xerophilous [20]. Examination of allozyme variation in *D. dahli* revealed the existence of five clones; however, unlike what was observed for the color varieties, one clone was widespread and abundant, and four other clones were rare and geographically restricted [18]. Based on such a pattern of allozyme clone distribution and the current allopatric occurrence of the parental bisexual species, these authors concluded that few bisexual individuals were involved in the formation of the *D. dahli* species, and that the initial successful clone was formed only once [18].

In our DNA fingerprinting studies of *D. dahli*, a rather high level of variation among individual fingerprint patterns was demonstrated when various microsatellite probes were used for blot hybridization [22,23]. However, the origin of this variation–whether it occurred as a result of point mutations or more complex genomic reorganization–remains obscure. New information regarding genetic variation and clone formation in *D. dahli* can be obtained via molecular and genetic studies of individual genomic loci.

The main objective of this study was to examine whether the *D. dahli* species originated from a single or multiple interspecific hybridization events. For this, we sequenced all allelic variants of three microsatellite loci found among 111 *D. dahli* specimens from five Armenian populations, identified nucleotide variations among sequenced alleles, and established a distinct genotype for each individual. We hypothesized that the nucleotide variations found among alleles in the hybrid genomes of *D. dahli* were inherited from two parental species, and that their specific combinations formed by two alleles in genotypes may reflect independent hybridization events between bisexual individuals of parental ancestors. In this way, we detected 11 genotypes and identified at least three hybridization founder events in the *D. dahli* populations sampled. These data provide new knowledge regarding the evolutionary origin of *D. dahli* that suggest its multiple hybridization origin. Our approach based on the detection of genotype-specific markers formed by combinations of parental-specific nucleotide markers can be used to address similar problems in other unisexual taxa.

Materials and Methods

Parthenogenetic lizards were collected between 1997 and 2006 from their natural habitats in Armenia. All territories in which lizard samples were collected are governmental. There are no protected areas or private land on these territories. Therefore, no specific permissions were required for our sample collections in these locations. Our field studies did not involve endangered or protected species, and according to the Red Book of Armenia, the genus *Darevskia* was not counted as an endangered species in that period [20]. The study was approved by the Ethics Committee of Moscow State University (Permit Number: 24-01) and was carried out in strict accordance with their ethical principles and scientific standards. Blood samples were taken from the tail veins of lizards under chloroform anesthesia, and all efforts were made to minimize suffering. DNA samples from lizard blood were isolated using a standard phenol–chloroform extraction method with proteinase K, and resuspended in TE buffer, pH 8.0. PCR amplification was performed using DNA samples from 111 *D. dahli* individuals of five isolated populations in Armenia that were available to us: "Papanino" ($n = 69$), "Fioletovo" ($n = 9$), "Vaagni" ($n = 17$), "Dzoraget" ($n = 6$), and "Dendropark" ($n = 10$) (Figure 1). The loci Du215, Du281, and Du323 were amplified using the primer pairs described previously (Du215:5′−CAACTAGCAG-TAGCTCTCCAGA−3′, 5′−CCAGACAGGCCCCAACTT−3′; Du281:5′−TTGCTAATCTGAATAACTG−3′, 5′−TCCTGCTGAGAAAGACCA−3′; Du323:5′−AAGCA-GACTGTACAAAATCCCTA−3′, 5′−ACTGATCTAAAGA-CAAGGTAAAAT−3′) [24,25]. These primers were first developed for the amplification of the respective loci in the parthenogenetic lizard species *D. unisexualis* [24]. In the course of cross-species amplification, they were found to be suitable for homeological loci amplification in the congeneric species *D. dahli*. The Du215, Du281, and Du323 loci were chosen for genotyping analysis because each of them was polymorphic and contained a tetranucleotide microsatellite cluster that met the requirements of good resolution of the individual allelic PCR products in a native polyacrylamide gel electrophoresis, thus allowing the direct sequencing of the individual alleles. In general, the procedure for the isolation and sequencing of individual alleles was carried out as described previously [24,25]. PCR was performed on 50 ng of DNA in a total volume of 20 μL using a GenePak PCR Core kit (Isogene, Russia) and 1 μM of each primer. The reaction conditions were: one cycle of 3 min at 94°C; 30 cycles of 1 min at 94°C; 40 s at the annealing temperature (58°C for Du215, 50°C for Du281, and 48°C for Du323); and 40 s at 72°C, followed by one cycle of 5 min at 72°C. PCR products (15 μL) were loaded onto an 8% native polyacrylamide gel (to separate allelic variants for each locus) and run for 12 h at 60 V. A 50 bp ladder (Fermentas) was used as a size marker. The amplification products were visualized by staining the DNA in the gel with ethidium bromide. The well-resolved individual PCR products, which corresponded to the two individual alleles of the locus, were excised from the gel, purified by ethanol reprecipitation, and sequenced directly in both directions using a chain termination reaction with an ABI PRISM BigDye Terminator ver. 3.1 on an Applied Biosystems 3730 DNA analyzer. This procedure was carried out twice for genotypes 5–11, because each of them was represented by only one individual. In all of these cases, allelic identity was checked and confirmed via the comparison of sequences obtained independently. In a separate experiment, the PCR product of the Du215 locus in genotype 11 was excised from the polyacrylamide gel, purified, and cloned into the pMos blue vector according to standard procedures (pMos Blue Blunt-ended

Cloning kit, RPN5110; Amersham Biosciences). The clones were amplified in MOSBlue competent cells grown at 37°C, and sequenced as described previously [25].

The nucleotide sequences were compared using the MEGA ver. 5.1 software [26]. All new data obtained from DNA sequencing were deposited in GenBank (FJ981592−FJ981598; FJ975076−FJ975081; FJ981559−FJ981561).

The number of alleles, allelic richness, as a measure of allele counts adjusted for sample size, and expected heterozygosity, as a measure of gene diversity, were calculated per locus and per population using the Fstat software ver. 2.9.3.2 [27].

The TCS program implements a statistical parsimony approach for estimating genealogical relationships between *D. dahli* genotypes using the method of Templeton *et al.* (1992) [28]. This method provides estimates of gene genealogy at the population level that are more accurate than traditional phylogenetic methods [29]. It is more frequently used for SNP comparison of maternally inherited mitochondrial genes, which are known for their neutrality and linear arrangement [30]. Usually, microsatellite repeats are also considered as evolutionarily neutral DNA markers [31]. Stable reproduction of the maternal diploid number of chromosomes in successive generations caused by premeiotic chromosome duplication during oogenesis in parthenogenetic lizards allowed us to consider homeological alleles at one locus as different genes, i.e., presentation of the two alleles of a heterozygous genotype in a linear arrangement. We assumed that the processes of gain and loss of microsatellite repeat units have an equal probability. Genotypes 1−9 were used for the raw matrix, in which each GATA repeat was designated by the letter A (Table S1). The program collapses identical sequences into haplotypes, calculates the frequencies of the haplotypes in the sample, and connects them into a network. Haplotype networks were established using TCS software ver. 1.21, with gaps being considered as a second state.

Results

In total, 111 specimens of *D. dahli* from five populations of Armenia were analyzed using locus-specific PCR and DNA sequencing of PCR amplificants. It was shown that all *D. dahli* individuals but one were heterozygous at the three loci analyzed, and contained two alleles that differed from each other regarding the length and structure of microsatellite clusters, and regarding single nucleotide variations (SNVs) in fixed positions of the flanking regions (Table 1). The number of alleles varied from three to seven (depending on the locus). All allelic variants of every locus could be divided into distinct groups according to the fixed nucleotide variations in the microsatellite flanking regions. For example (Table 1), fixed nucleotide variations in alleles 1–4 of Du215 formed the SNV set TGC, whereas those in alleles 5 and 6 of Du215 formed the SNV set ACC, and those in allele 7 of Du215 formed the SNV set ACT.

To identify genotypic diversity in *D. dahli* populations, allelic combinations of the three loci in each of the 111 individuals were constructed. In total, 11 genotypes that differed in population frequencies and geographical distribution were revealed (Table 2). The individuals with identical genotypes formed distinct clonal lineages. One clone (genotype 1) was abundant (designated as major) and was represented by 72 individuals (64.9% of the total cohort) in three sampled populations; another (genotype 2) was less abundant (designated as intermediate) and was represented by 21 individuals (18.9% of the total cohort) in four populations; nine

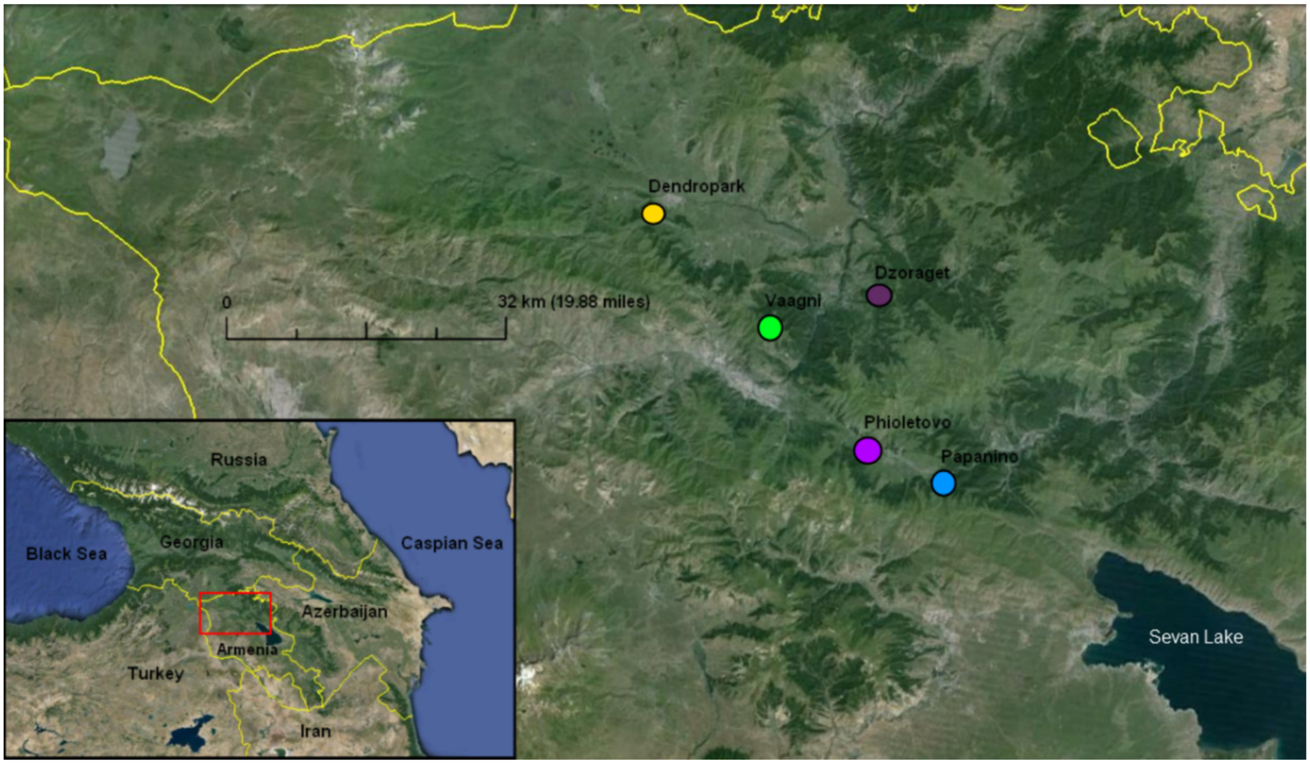

Figure 1. Map of Armenia with the distribution of localities from which parthenogenetic *Darevskia dahli* populations were collected. Sampling localities are indicated by different colours. Papanino (+40° 42′ 27.76″, +44° 45′ 43.89″), Phioletovo (+40° 44′ 29.53″, +44° 39′ 31.25″), Vaagni (+40° 52′ 19.16″, +44° 31′ 28.60″), Dzoraget (+40° 54′ 15.02″, +44° 40′ 37.78″), Dendropark (+40° 59′ 30.55″, +44° 21′ 50.79″).
doi:10.1371/journal.pone.0091674.g001

Table 1. Allelic variations of microsatellite containing loci Du215, Du281, and Du323 in parthenogenetic *D. dahli* species.

Allelic variant	Size (bp)	Structure of microsatellite cluster	Fixed nucleotide variations[1]	EMBL/Gene Bank ac. No.	Source
Du215(dahli)1	252	5′ (GAT)(GACA)(GATA)$_{10}$(GACA)$_7$(GATA)(GCAA) 3′	T (−58), G (−38), C (−19)	FJ981592	this study
Du215(dahli)2	248	5′ (GAT)(GACA)(GATA)$_9$ (GACA)$_7$(GATA)(GCAA) 3′	T (−58), G (−38), C (−19)	FJ981593	
Du215(dahli)3	244	5′ (GAT)(GACA)(GATA)$_8$ (GACA)$_7$(GATA)(GCAA) 3′	T (−58), G (−38), C (−19)	FJ981594	
Du215(dahli)4	232	5′ (GAT)(GACA)(GATA)$_7$ (GACA)$_5$(GATA)(GCAA) 3′	T (−58), G (−38), C (−19)	FJ981595	
Du215(dahli)5	232	5′ (GAT)(GATA)$_{11}$(GCAA)$_4$ 3′	A (−58), C (−38), C (−19)	FJ981596	
Du215(dahli)6	228	5′ (GAT)(GATA)$_{10}$(GCAA)$_4$ 3′	A (−58),C (−38), C (−19)	FJ981597	
Du215(dahli)7	192	5′ (GAT)(GATA)$_5$ 3′	A (−58), C (−38), T (−19)	FJ981598	
Du281(dahli)1	225	5′ (GGTA)(GATA)$_9$(GAT)(GATA)(GGTA)$_2$(GAT)(GATA)$_4$ 3′	T (+15)	FJ975076	Davoyan et al. [42]
Du281(dahli)2	221	5′ (GGTA)(GATA)$_8$(GAT)(GATA)(GGTA)$_2$(GAT)(GATA)$_4$ 3′	T (+15)	FJ975077	this study
Du281(dahli)3	199	5′ (GATA)$_{12}$ 3′	C (+15)	FJ975078	this study
Du281(dahli)4	195	5′ (GATA)$_{11}$ 3′	C (+15)	FJ975079	Davoyan et al. [42]
Du281(dahli)5	191	5′ (GATA)$_{10}$ 3′	C (+15)	FJ975080	
Du281(dahli)6	183	5′ (GATA)$_8$ 3′	C (+15)	FJ975081	this study
Du323(dahli)1	215	5′ (AC)$_6$…(GATA)$_{11}$(GAT)(GATA)(GATATAT)(GA)$_4$ 3′	C (−23), T (+39)	FJ981559	Vergun et al. [43]
Du323(dahli)2	211	5′ (AC)$_6$…(GATA)$_{10}$(GAT)(GATA)(GATATAT)(GA)$_4$ 3′	C (−23), T (+39)	FJ981560	
Du323(dahli)3	184	5′ (AC)$_5$…(GATA)(GGT)(GATA)$_3$(GAT)(GATATAT)(GA)$_4$ 3′	A (−23), C (+39)	FJ981561	

[1]Distances before (−) and after (+) microsatellite cluster are given in bp.
doi:10.1371/journal.pone.0091674.t001

clones were rare, geographically restricted, and were represented by one (genotypes 5–11; each in 0.9% of the total cohort) or six and five (genotypes 3 and 4, 5.4% and 4.5% of the total cohort, respectively) individuals.

One can see that the genotypic diversity among the five lizard populations varied from 0% to 44.4% (average, 9.9%) (Table 2). The highest levels of genotypic diversity were observed in the Vaagni and Dendropark populations. We calculated the number of population genetic indices for k loci using the Fstat software (Table S2). The genetic variations of genotypes 1−9 were used. The estimates of expected heterozygosity varied from 0.51 to 0.65 (average, 0.56). The number of alleles varied from 2 to four with 2.7 in average. The values of allelic richness varied from 2.000 to 3.453 with 2.570 in average. The highest allelic scores, the number of alleles as well as allelic richness (Rs) were observed in two populations for the all three loci Dendropark and Vaagni: 3.453 and 2.938, respectively (Du215), 2.600 and 2.800, respectively (Du281), 2.853 and 2.648, respectively (Du323).

To clarify whether the *D. dahli* species originated from single or multiple interspecies hybridization events, genotype-specific nucleotide markers formed by combinations of parental-specific SNVs of three loci were established and compared. Figure 2 schematically shows the structural composition of 11 genotypes that included microsatellite clusters and parental-specific SNVs in fixed positions in the flanking regions (Table 1). It was clear that genotypes 1–9 had identical combinations of parental-specific SNVs for all three loci: TGC/ACC (Du215), T/C (Du281), and CT/AC (Du323). This means that they have identical genotype-specific markers and, therefore, may have a common origin as the result of a single interspecies hybridization event. However, the possibility of independent crossings of the parental individuals with frequent respective allelic variants for some of these genotypes cannot be ruled out. Genotypes 10 and 11 differ from each other and from genotypes 1–9 by parental-specific SNV combinations for locus Du215: TGC/ACT and TGC/TGC, respectively. Thus, we suggest that genotypes 10 and 11 have independent origins. Consequently, at least three independent interspecific hybridization events took place in the genesis of the 11 genotypes. Several additional lines of evidence suggest that the microsatellite clones observed in *D. dahli* result from at least three independent interspecies hybridization events, rather than from a single origin. In addition to having the same combination of parental-specific SNVs, genotypes 1–9 also exhibited a similar organization of allelic microsatellite clusters. Different situations occurred in individuals with genotypes 10 and 11. The microsatellite cluster of the ACT allele of genotype 10, unlike what was observed for all other alleles, had no (GCAA)$_4$ nucleotide group. The individual with genotype 11, unlike all other individuals, was homozygous at the Du215 locus.

An unresolved question remains regarding the relationships between genotypes 1–9. These genotypes differed from each other only by microsatellite sequences. Figure 3 schematically shows the resulting SP network, which reflects the relationships between genotypes 1–9 (genotypes 10 and 11 are plotted separately). The areas of the circles are correlated with the abundance of the genotype. The areas of the circle segments are correlated with the population distribution for each genotype. The black circles show, unsampled but computer-predicted genotypes that participated in the formation of genotypes 5, 6, and 8.

According to Figure 3, the most distant genotypes were unique genotypes 6 and 5 from Papanino and Vaagni, respectively. It should be noted that the maximum variability of genotypes was observed in the latter population (genotypes 1, 2, 4, 5, and 8−11). Three of them (5, 8, and 9) were specific for the Vaagni population. Two genotypes (3 and 7) were specific for the Dendropark population among the three genotypes discovered. Only genotype 6 of the four genotypes detected was specific in the Papanino population, and no specific genotypes were found in the Fioletovo and Dzoraget populations. Genotypes 4, 1, and 2 were identified in two, three, and four populations, respectively. The analysis of the spatial-frequency distributions of the three more widely distributed genotypes and of a few population-specific

Table 2. Sample size, combined genotype structure, diversity and distribution in the *D. dahli* populations.

Genotype	Allelic combination	Populations					Number of individuals with definite genotype (genotype frequencies)
		Pa	Ph	Va	Dz	De	
1	Du215(3+6)+Du281(1+4)+Du323(1+3)	61	9	2	0	0	72 (0,649)
2	Du215(3+6)+Du281(1+4)+Du323(2+3)	6	0	7	6	2	21 (0,189)
3	Du215(2+6)+Du281(1+4)+Du323(1+3)	0	0	0	0	6	6 (0,054)
4	Du215(3+5)+Du281(1+4)+Du323(2+3)	1	0	4	0	0	5 (0,045)
5	Du215(3+5)+Du281(2+4)+Du323(2+3)	0	0	1	0	0	1 (0,009)
6	Du215(3+6)+Du281(1+6)+Du323(1+3)	1	0	0	0	0	1 (0,009)
7	Du215(1+6)+Du281(1+4)+Du323(1+3)	0	0	0	0	1	1 (0,009)
8	Du215(2+6)+Du281(1+5)+Du323(1+3)	0	0	1	0	0	1 (0,009)
9	Du215(3+6)+Du281(1+3)+Du323(2+3)	0	0	1	0	0	1 (0,009)
10	Du215(4+7)+Du281(1+6)+Du323(2+3)	0	0	1	0	0	1 (0,009)
11	Du215(3)+Du281(1+4)+Du323(2+3)	0	0	1	0	0	1 (0,009)
Total number of individuals		69	9	18	6	9	111
Genotype diversity (%)		4 (5,8)	1 (0)	8 (44,4)	1 (0)	3 (33,4)	11 (9,9)

Pa – Papanino.
Ph – Phioletovo.
Va – Vaagni.
Dz – Dzoraget.
De – Dendropark.
doi:10.1371/journal.pone.0091674.t002

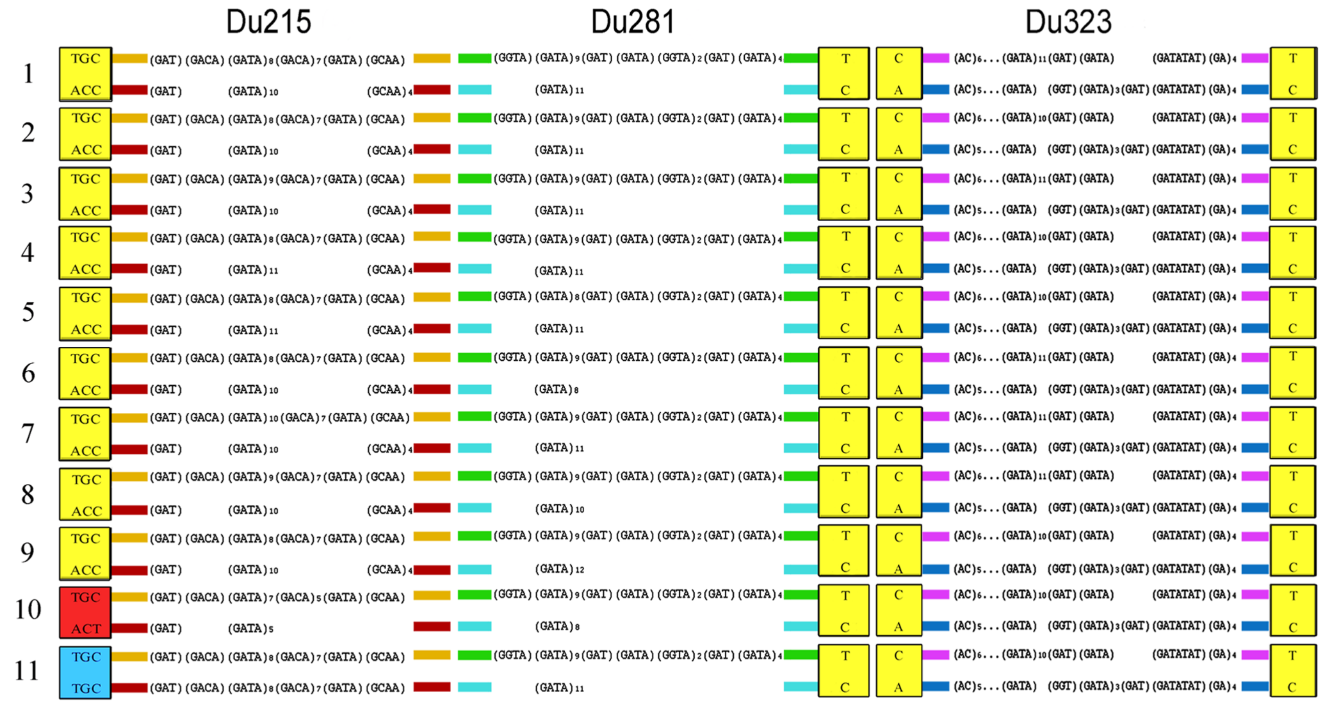

Figure 2. Schematic representation of 11 genotypes formed by allelic combinations of microsatellite loci Du215, Du281, and Du323 in 111 *D. dahli* individuals. Parental-specific SNV markers are shown in square. Variable microsatellite clusters are shown in each of two alleles.
doi:10.1371/journal.pone.0091674.g002

genotypes revealed a dependence between the frequencies of the clones and the geographical disjunction between the Vaagni population and two groups of populations, Papanino/Fioletovo and Dzoraget/Dendropark. Namely, genotypes 1, 2, 4, and 6 were discovered in the Papanino and Fioletovo populations, which are located at a distance of 9.5 km from each other. Genotypes 2, 3, and 7 were distributed in the Dzoraget and Dendropark populations, which are located at a distance of 28 km from each other. It follows that genotype 2 was shared by the two population groups. First, in the Vaagni population, which is located closer to the Dzoraget/Dendropark group (13−19 km) than it is to the Papanino/Fioletovo populations (18−27 km), the common genotype 2 was present, as were genotypes 1 and 4, which were also discovered in the Papanino/Fioletovo populations. Second, genotype 8 was discovered only in the Vaagni population and can be regarded as a derivative from genotype 3, which was specific to the Dzoraget/Dendropark group. Thus, the Vaagni population might be the source of the clonal diversity observed among the five populations studied.

Discussion

The main finding of this study was the presence of at least three interspecific hybridization founder events in *D. dahli*, which resulted in the formation of three initial clones and eight clones that probably arose through postformation microsatellite mutations of the initial clones. Previously, an allozyme analysis conducted at 35 gene loci in three Armenian and three Georgian sampled populations of *D. dahli* revealed one major widespread clone and four rare clones detected only in restricted populations [18]. The authors concluded that the observed allozyme pattern in *D. dahli* generally followed Parker *et al.*'s model [10], which is consistent with its single hybridization origin. Nevertheless, they noted that possible multiple origins for *D. dahli* should be investigated further using more effective genomic markers [18]. Among parthenogenetic *Darevskia*, *D. rostombekovi* was also suggested to be of single origin [32]. In *D. armeniaca*, four allozyme clones were detected [33], but one rare clone made up the majority of two populations, and another rare clone had different allelic compositions at two loci. The authors assumed that both mutation and multiple origin could be alternatively considered as a possible explanation of the origin of the clonal variation in *D. armeniaca*. However, the allozyme data failed to distinguish multiple origins and mutation events. Fu *et al.* (2000) noted that many studies that were performed at the species level examined various population characteristics, but few did this at the genotype level [33].

In our study, a new approach for the detection of *D. dahli* genotypes formed by independent hybridization between bisexual parental individuals was developed. This approach included microsatellite genotyping of the sampled individuals, and the identification of genotype-specific nucleotide markers formed by combinations of parental-specific nucleotide markers (SNVs). The examination of the allelic combinations of three microsatellite loci in 111 *D. dahli* individuals revealed 11 clones, two abundant (major and intermediate) and nine rare and geographically restricted. This pattern of microsatellite-clone distribution raises questions about the scenario of the *D. dahli* species formation, and about the relationship between major, intermediate, and rare clones. The comparison of genotype-specific markers showed that all individuals with genotypes 1–9 exhibited the same combination of parental-specific SNVs and, therefore, could have a common origin. Two individuals with genotypes 10 and 11, respectively, possessed a unique combination of parental-specific SNVs, and thus were likely to have independent origins. Our data do not rule out further hybridization events. First, the variation observed among clones 1−9 could actually be a polymorphism that was inherited from the parental populations, and there were differences between individuals with similar genotypes; that is one

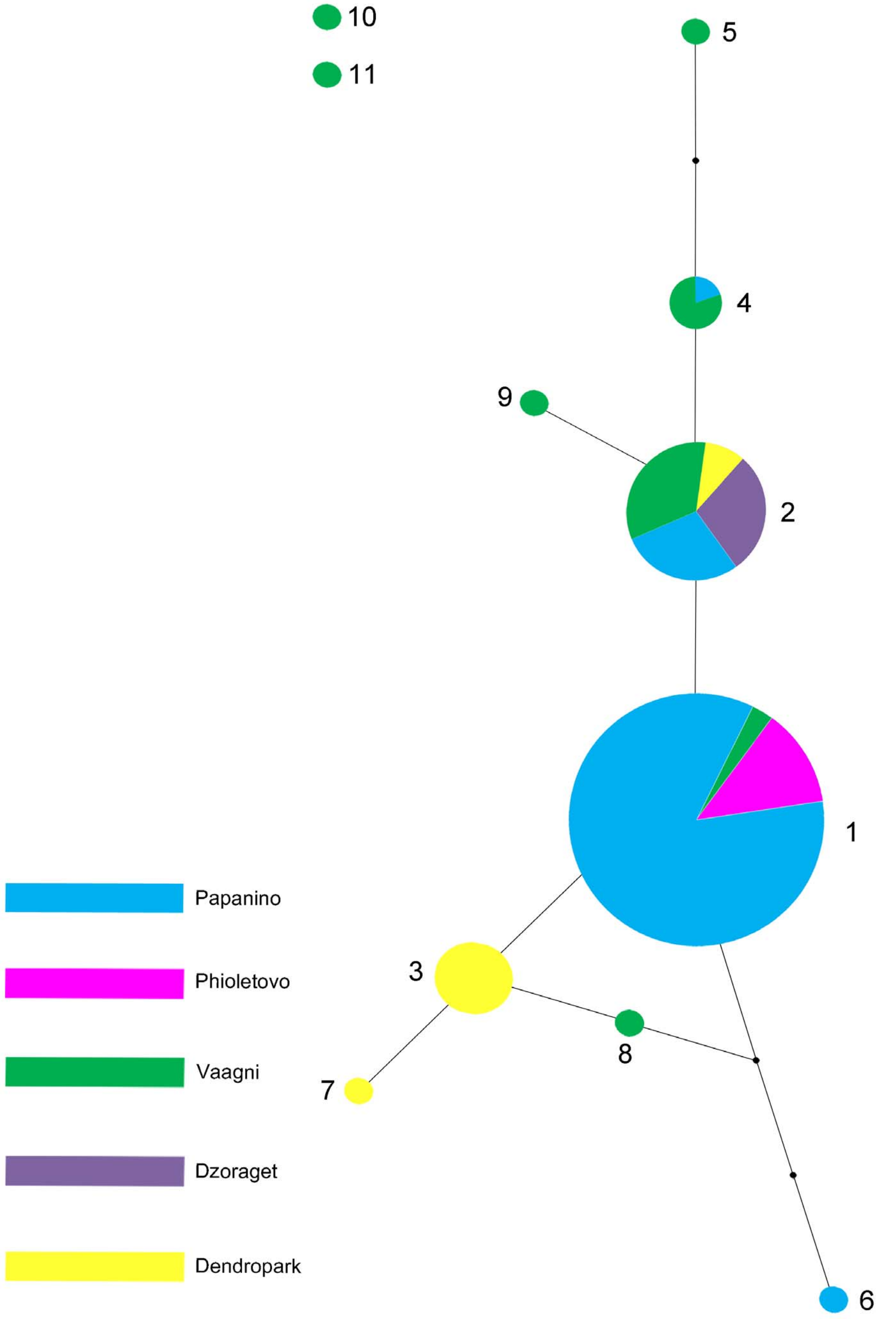

Figure 3. Schematic representation of the SP network that reflects relationship between genotypes 1–9 in *D. dahli.* Complete sequences of *D. dahli* genotypes were analyzed using TCS software version 1.21. Genotypes 10 and 11 are plotted separately. Population distribution of the genotypes is shown by different colours. The black circles show, unsampled, but computer-predicted genotypes.
doi:10.1371/journal.pone.0091674.g003

potential source of underestimation of the frequency of hybridization. Second, in the case of low variation in parental populations, some hybridization events could have led to identical unisexual genotypes with fewer hybridization events. Nevertheless, some of the clones 1−9 may indeed have originated via postformation microsatellite mutations of the initial clones. Consideration of the SP network, which reflects the relationships between genotypes 1–9, does not allow the disclosure of the initial clone. According to Parker *et al.*'s model [10], we predicted that the major widespread clone with genotype 1 could be the initial clone, whether all others probably arose through postformation microsatellite mutations of the major clone. Figure 3 shows that from one to four mutational events led to the formation of new microsatellite clones. Previously, we characterized the highly unstable $(GATA)_n$-containing locus in parthenogenetic *D. unisexualis*. We identified and sequenced mutant alleles in some offspring of the first generation and showed that *de novo* mutations occurred via deletion or insertion of a single microsatellite repeat [25].

Multiple hybridization events leading to multiple allozyme clones have been described in *Cnemidophorus* and *Heteronotia* [34]. The patterns of allozyme variability among species of multiple origins ranged from a large variation (*Heteronotia binoei*; [6]) to

medium (*Cnemidophorus tesselatus*; [35]), and slight (*Darevskia* parthenogens; [18,32]) variations. Thus, the patterns of allozyme variation in *D. dahli* [18] do not contradict the multiple hybridization origin demonstrated in our study, and the higher microsatellite clonal diversity reflects the higher effectiveness of the markers used.

Nine relatively rare clones, each represented by one or several individuals, were found in four sampled populations of *D. dahli*. Of the rare clones, six, including those with genotypes 10 and 11, occurred in the Vaagni population. This population, together with the Dendropark population, has an enhanced level of clonal diversity (Table 2, Figure 3). Interpopulation differences formed by the distribution of various genotypes can be explained by the distinct ecological conditions of the habitat of *D. dahli* populations. However, differences in suitable landscapes, rock dimensions, and the resource utilization curves are secondary for *D. dahli* with respect to the differences regarding preferred climates [36]. This species has a narrow climatic niche and its distribution depends mainly on both temperature and humidity. Thus, the varied topography, vegetation, and climate can influence some disjunct distribution and interpopulation differences of *D. dahli* populations. Some other factors, which are connected with the frequency of interspecific mating at some localities and hybrid fitness ability, could also be significant in the case of the sympatric existence of the parental bisexual species in the past. At present, only one of the studied *D. dahli* populations (Dzoraget) overlaps with the bisexual parental species *D. portschinskii*, but the upper altitudinal limit of the unisexual species is higher [19]. The interaction between *D. dahli* and *D. portschinskii* is restricted to competition for food or cover and, as noted above, the interspecific hybrids observed were infertile triploids [21].

The greater clonal diversity of the Vaagni and Dendropark populations could be also connected with their geographical location in the seismically active region of Armenia, where a casting of chemical mutagens has sometimes occurred [37]. Under these conditions, the accumulation of microsatellite mutations might be increased. In previous studies, a high level of chromosomal instability was observed in some rodent and lizard species from this region, as well as chromosomal speciation events [38−41].

The lack of statistical approaches for the evaluation of population diversity in parthenogenetic species has forced us to use parsimony networks, which are well known in the field of phylogeography, to demonstrate the peculiarities of the geographical distributions of individual clones. Despite the formal similarity of the two types of molecular markers, the mechanisms of accumulation of nucleotide substitutions (SNPs) in mitochondrial DNA molecules are rather different. Here, we used many simplifications and assumptions, such as the absence of linkage among the three loci, the independence of replication events at each of the three loci, the equal rate of microsatellite repeat deletions or insertions, and the lack of intergenic recombination and genic and chromosomal interference [31]. These processes can distort the relationships between the genotypes; hence, in the course of parsimony network analysis, we evaluated only peculiarities in the geographical distributions of the genotypes. Therefore, we were not able to assess the possible time of divergence and specify the ancestral clone.

Low intraspecific variation and a high degree of mtDNA similarity regarding the parental species have been a general rule in parthenogenetic *Darevskia* species [6]. These features have been explained by young age. Darevsky *et al.* (1985) estimated that the parthenogenetic Caucasian rock lizards originated after the Pleistocene glaciation of the Caucasian mountains, 5000 to 7000 years ago [16]. Based on the allozyme analysis of *D. dahli* and two parental species, Murphy *et al.* [18] suggested the possibility that *D. dahli* arose in Georgia and dispersed into Armenia. This is correlated with today's Georgian localization of the maternal species *D. mixta* and the strong allopatric distribution of parental species. It can also mean that the initial microsatellite clones detected by us originated in Georgia, and then dispersed to Armenia. At the same time, the possibility that these clones originated in Armenia could not be excluded, because many climatic and vegetative changes have occurred postglacially in the Caucasus region, providing an abundance of microhabitats for lizard species. An alternative scenario of the origin of *D. dahli* can also be considered. Two populations, Vaagni and Dendropark, were characterized by the highest estimates of genetic diversity (Table S2). However, in the case of sympatric distribution of the parental species in the past, the most probable *D. dahli* origin should be the site of the Vaagni population. The greatest number of genotypes, including six specific genotypes (4, 5, and 8–11) was detected in this population (Figure 3, Table 2). Moreover, the two of them, genotypes 10 and 11, originated from independent bisexual parental individuals. This scenario supposes the further distribution of *D. dahli* from the *Vaagni* site into other locations in Armenia. Clone 5 might be one of the putative initial clones, according to the scheme (Figure 3). Genotypes 4, 2, 9, and 1, which are derivatives of clone 5, originated in the course of lizard migration in a northern direction (Vaagni to Dendropark), as well as in a southern direction (Dzoraget to Papanino, to Fioletovo). In the latter case, the river Pambak may be a geographical barrier for lizard migrations, which is the reason for the low genetic diversity and abundance of clone 1 in the Papanino/Fioletovo populations. However, the proposed scenario is not the only one. Regardless of where the initial clones were originated, their abundance as well as that of clones originated through microsatellite mutations may be correlated with a climatic dissimilarity of the habitats [36]. All mutant clones should have a later origin relating to the initial clones. Among them, under similar ecological conditions, more abundant clones probably arose first.

The results obtained for population genetic parameters may be useful for comparing the samples studied with the parental species. Moreover, the estimates of population genetic indices may be useful when comparing parthenogenetic reptiles belonging to different genera.

It should be noted that our microsatellite genotyping data, as well as allozyme [18] and mtDNA [8] studies, failed to distinguish the two color varieties ("yellow" and "common" morphological variants) of *D. dahli*. Among the 111 individuals of *D. dahli*, only 14 belong to the "yellow" morphological variant. All these lizards were found in the Papanino population and had genotype 1. We believe that two morphological variants of *D. dahli* are genetically determined because they also differ functionally, namely in egg production. Further research using new microsatellite loci will probably differentiate these forms.

In summary, our data suggest that the clonal diversity observed for *D. dahli* derives from both multiple interspecific hybridization origins and postformation microsatellite mutations of the initial clones. The methodological approach developed in this study, which was based on the detection of genotype-specific markers formed by combinations of parental-specific markers, can be further applied for the elucidation of the origin and evolution of unisexual species.

Supporting Information

Table S1 Polymorphisms of three microsatellite loci Du215, Du281, and Du323 for nine genotypes in *D. dahli* (see Figure 2). GATA repeat is designated by the letter A. Numbers before the letter A designate the number of the repeat on each of the two chromosomes that are separated in the Table by forward slash "/". In concatenated sequences used for parsimonies network reconstruction, the absence of a GATA repeat is designated by dash (−).
(DOC)

Table S2 The population indices of gene diversity for three studied loci in five sampled populations. N – number of alleles, Rs – allelic richness, H_E – expected heterozygosity.
(DOC)

Author Contributions

Conceived and designed the experiments: APR ONT. Performed the experiments: AAV IAM. Analyzed the data: AAV VIK SKS AVO VGP OEL. Contributed reagents/materials/analysis tools: APR. Wrote the paper: APR.

References

1. Sinclair EA, Pramuk JB, Bezy RL, Crandall KA, Sites JW (2010) DNA evidence for nonhybrid origins of parthenogenesis in natural populations of vertebrates. Evolution 64(5): 1346–1357.
2. Vrijenhoek RC, Dawley RM, Cole CJ, Bogart JP (1989) A list of known unisexual vertebrates. In: Dawley RM, Bogart JP, editors. Evolution and Ecology of Unisexual vertebrates. Albany, N.Y.: New York State Museum Bulletin 466. 19−23.
3. Darevsky IS (1958) Natural parthenogenesis in certain subspecies of rock lizards (*Lacerta saxicola* Eversmann). Dokl Akad Nauk SSSR Biol Sci 122: 730−732.
4. Simon JC, Delmotte F, Rispe C, Crease T (2003) Phylogenetic relationships between parthenogens and their sexual relatives: the possible routes to parthenogenesis in animals. Biol J Linn Soc Lond 79: 151−163.
5. Cole CJ, Dessauer HC, Barrowclough GF (1988) Hybrid origin of a unisexual species of whiptail lizard, *Cnemidophorus neomexicanus*, in western North America: new evidence and a review. Am Mus Novit 2905: 1–38.
6. Moritz C, Donnelan S, Adams M, Baverstock PR (1989) The origin and evolution of parthenogenesis in *Heteronotia binoei* (Gekkonidae): Extensive genotypic diversity among parthenogens. Evolution 43: 994–1003.
7. Parker ED (1979) Ecological implications of clonal diversity in parthenogenetic morphospecies. Am Zool 19: 753–762.
8. Moritz C, Uzzel T, Spolsky C, Hotz H, Darevsky IS, et al. (1992) The maternal ancestry and approximate age of parthenogenetic species of Caucasian rock lizards (Lacerta: Lacertidae). Genetica 87: 53−62.
9. Dessauer HC, Cole CJ (1989) Diversity between and within nominal forms of unisexual teiid lizards. In: Dawley RM, Bogart JP, editors. Evolution and Ecology of Unisexual vertebrates. Albany, N.Y.: New York State Museum Bulletin 466. 49−71.
10. Parker ED, Walker JM, Paulissen MA (1989) Clonal diversity in *Cnemidophorus*: ecological and morphological consequences. In: Dawley RM, Bogart JP, editors. Evolution and Ecology of Unisexual vertebrates. Albany, N.Y.: New York State Museum Bulletin 466. 72−86.
11. Densmore LD, Moritz C, Wright JW, Brown WM (1989) Mitochondrial DNA analysis and the origin and relative age of parthenogenetic lizards (Genus *Cnemidophorus*) IV. Nine *sexlineatus* group unisexualis. Evolution 43: 469−483.
12. Vyas DK, Moritz C, Peccinini-Seale D, Wright JW, Brown WM (1990) The evolutionary history of parthenogenetic *Cnemidophorus lemniscatus* (Sauria, Teiidae). II. Maternal origins inferred from mitochondrial DNA analysis. Evolution 44: 922−932.
13. Moritz C (1991) The origin and evolution of parthenogenesis in *Heteronotia binoei*: Evidence for recent and localized origins of widespread clones. Genetics 129: 211−219.
14. Parker ED, Selender RK (1984) Low clonal diversity in the parthenogenetic lizard *Cnemidophorus neomexicanus* (Sauria: Teiidae). Herpetologica 40: 245−252.
15. Densmore LD, Wright JW, Brown WM (1989) Mitochondrial DNA analysis and the origin and relative age of parthenogenetic lizards (Genus *Cnemidophorus*) II. *C. neomexicanus* and the *C. tesselatus* complex. Evolution 43: 943−957.
16. Darevsky IS, Kupriyanova LA, Uzzel T (1985) Parthenogenesis in Reptiles. In: Gans C, Billett DF, editors. Biology of Reptilia. 15. New York: John Wiley & Sons. 413–526.
17. Uzzell TM, Darevsky IS (1975) Biochemical evidence for the hybrid origin of the parthenogenetic species of the *Lacerta saxicola* complex (Sauria, Lacertidae), with a discussion of some ecological and evolutionary implications. Copeia 2: 204−222.
18. Murphy RW, Darevsky IS, MacCulloch RD, Fu J, Kupriyanova LA, et al. (1997) Olde age, multiple formations or genetic plasticity? Clonal diversity in the uniparental Caucasian rock lizard, *Lacerta dahli*. Genetica 101: 125−130.
19. Darevsky IS (1967) Rock lizards of the Caucasus: systematics, ecology and phylogenesis of the polymorphic groups of Caucasian rock lizards of the subgenus *Archaeolacerta*. Leningrad: Nauka, 1−216 [in Russian: English translation published by Indian Natl Sci Doc Ctr, New Delhi, 1978].
20. Arakelyan MS, Danielyan FD, Corti C, Sindaco R, Leviton AE (2011) Herpetofauna of Armenia and Nagoro-Karabakh. Salt Lake City: Society for Study of Amphibians and Reptiles. 154 p.
21. Davoyan A (2007) Study of morphological and genetic variability of parthenogenetic rock lizard *Darevskia dahli*. Yerevan, Armenia: PhD Thesis. 119 p.
22. Kan NG, Petrosyan VG, Martirosyan IA, Ryskov AP, Darevsky IS, et al. (1998) Genomic Polymorphism of mini- and microsatellite loci of the parthenogenic *Lacerta dahli* revealed by DNA Fingerprinting. Mol Biol 32(5): 672−678.
23. Ryskov AP (2008) Genetically unstable microsatellite-containing loci and genome diversity in clonally reproduced unisexual vertebrates. In: Jeon KW, editor. International Review of Cell and Molecular Biology. 270. USA: Academic Press. 319–349.
24. Korchagin VI, Badaeva TN, Tokarskaya ON, Martirosyan IA, Darevsky IS, et al. (2007) Molecular characterization of allelic variants of $(GATA)_n$ microsatellite loci in parthenogenetic lizards *Darevskia unisexualis* (Lacertidae). Gene 392: 126−133.
25. Badaeva TN, Malysheva DN, Korchagin VI, Ryskov AP (2008) Genetic variation and *de novo* mutations in the parthenogenetic Caucasian rock lizard *Darevskia unisexualis*. PLoS ONE 3(7): e2730. doi:10.1371/journal.pone.0002730.
26. Tamura K, Peterson D, Peterson N, Stecher G, Nei M, et al. (2011) MEGA5: molecular evolutionary genetics analysis using maximum likelihood, evolutionary distance, and maximum parsimony methods. Mol Biol Evol 28(10): 2731–2739.
27. Goudet J (1995) FSTAT (Version 1.2): a computer program to calculate F-statistics. J Hered 86(6): 485–486.
28. Templeton AR, Crandall KA, Sing CF (1992) A cladistic analysis of phenotypic associations with haplotypes inferred from restriction endonuclease mapping and DNA sequence data. III. Cladogram estimation. Genetics 132(2): 619–633.
29. Clement M, Posada D, Crandall KA (2000) TCS: a computer program to estimate gene genealogies. Mol Ecol 9(10): 1657–1659.
30. Posada D, Crandall KA (2001) Intraspecific gene genealogies: trees grafting into networks. Trends Ecol Evol 16(1): 37–45.
31. Ellegren H (2004) Microsatellites: simple sequences with complex evolution. Nat Rev Genet 5: 435–445.
32. MacCulloch RD, Murphy RW, Kupriyanova LA, Darevsky IS (1997) The Caucasian rock lizard *Lacerta rostombekovi*: a monoclonal parthenogenetic vertebrate. Biochem Syst Ecol 25: 33–37.
33. Fu J, MacCulloch RD, Murphy RW, Darevsky IS (2000) Clonal variations in the Caucasian rock lizard *Lacerta armeniaca* and its origin. Amphib-reptil 21(1): 83–89.
34. Moritz C, Brown WM, Densmore LD, Wright JW, Vyas D, et al. (1989) Genetic diversity and the dynamics of hybrid parthenogenesis in *Cnemidophorus* (Teiidae) and *Heteronotia* (Gekkonidae). In: Dawley RM, Bogart JP, editors. Evolution and Ecology of Unisexual vertebrates. Albany, N.Y.: New York State Museum Bulletin 466. 87−112.
35. Parker ED, Selander RK (1976) The organization of genetic diversity in the parthenogenetic lizard *Cnemidophorus tesselatus*. Genetics 84: 791–805.
36. Tarkhnishvili D, Gavashelishvili A, Avaliani A, Murtskhvaladze M, Mumladze L (2010) Unisexual rock lizard might be outcompeting its bisexual progenitors in the Caucasus. Biol J Linn Soc Lond 101: 447−460.
37. Karakhanian A, Djrbaschian R, Trifonov V, Philip H, Arakelian S, et al. (2002) Holocene-historical volcanism and active faults as natural risk factors for Armenia and adjacent countries. Journal of Volcanology and Geothermal Research 113: 319−344.
38. Savic I, Soldatovic B (1979) Distribution range and evolution of chromosomal forms in the Spalacidae of the Balkan Peninsula and bordering regions. J Biogeogr 6: 363−374.
39. Wahrman J, Goitein R, Nevo B (1969) Mole rat *Spalax*: evolutionary significance of chromosome variation. Science 164: 82.
40. Lyapunova EA, Ahverdyan MR, Vorontsov NN (1988) Robertson's fan of variability of chromosome in Caucasian subalpic field-vole (*Pitymys*, Microtinae, Rodentia). Russ J Genet 9(2): 480−483.
41. Kupriyanova LA (1974) Additional chromosomes of *Mabuya aurata septemtaeniata* lizard. Tsitologiya i Genetika 16(12): 1519−1521.
42. Davoyan AG, Aslanyan AV, Danielyan FD, Darevsky IS, Martirosyan IA (2007) Revealing of allelic polymorphism in the populations of parthenogenetic lizards *Darevskia dahli* (Lacertidae) using locus-specific PCR. Russ J Genet 43(1): 20–23.
43. Vergun AA, Markelova ES, Martirosyan IA (2007) Molecular-genetic characteristics of the Du323 locus containing various microsatellite types in the parthenogenetic lizard *Darevskia dahli* (Lacertidae). Dokl Biochem Biophys 416: 278−280.
